# Pediatric lawn mower-related injuries and contributing factors for bystander injuries

**DOI:** 10.1186/s40621-023-00468-z

**Published:** 2023-10-20

**Authors:** Charles A. Jennissen, Treyton D. Krupp, J. Priyanka Vakkalanka, Pamela J. Hoogerwerf

**Affiliations:** 1 Department of Emergency Medicine, Roy J. and Lucille A. Carver College of Medicine, University of Iowa, Iowa City, IA USA; 2 Stead Family Department of Pediatrics, Roy J. and Lucille A. Carver College of Medicine, University of Iowa, Iowa City, IA USA; 3 Roy J. and Lucille A. Carver College of Medicine, University of Iowa, Iowa City, IA USA; 4 Injury Prevention and Community Outreach, University of Iowa Stead Family Children’s Hospital, University of Iowa, Iowa City, IA USA

**Keywords:** Amputation, Backover, Bystander, Children, Injury, Limb loss, Lawn mower, Passenger, Pediatrics, Prevention, Reverse, Rides, Safety, Supervision

## Abstract

**Background:**

Riding lawn mower injuries are the most common cause of major limb loss in young U.S. children. Our study objective was to investigate the circumstances surrounding pediatric riding lawn mower injuries and to identify potential contributing risk factors and behaviors leading to these events.

**Methods:**

Followers/members of both a public and a private lawn mower injury support and prevention Facebook page who had or were aware of children who had suffered a lawn mower-related injury were invited to complete an electronic survey on Qualtrics. Duplicate cases and those involving push mowers were removed. Frequencies and chi-square analyses were performed.

**Results:**

140 injured children were identified with 71% of surveys completed by parents and 19% by an adult survivor of a childhood incident. The majority of injured children were Caucasian (94%), male (64%), and ≤ 5 years of age at the time of the incident (63%). Bystanders were 69% of those injured, 24% were lawn mower riders, and mower operators and others accounted for 7%. The lawn mower operator was usually male (77%), being the father/stepfather in almost half. Overall, 59% of injuries occurred while traveling in reverse, 29% while moving forward. Nearly all (92%) had an amputation and/or permanent disability. Subgroup analysis (*n* = 130) found injured bystanders were younger than injured passengers with 71% versus 45% being < 5 years of age, respectively (*p* = 0.01). Over three-quarters of bystander incidents occurred while moving in reverse as compared to 17% of passenger incidents (*p* < 0.01). Amputations and/or permanent disabilities were greater among bystanders (97%) as compared to passengers (79%, *p* = 0.01). Only 3% of bystanders had an upper extremity injury as compared to 21% of passengers (*p* = 0.01). Seventy-three percent of bystander victims had received at least one ride on a lawn mower prior to their injury incident.

**Conclusions:**

Child bystanders seriously injured by riding lawn mowers were frequently given prior rides likely desensitizing them to their inherent dangers and leading them to seek rides when mowers were being used. Engineering changes preventing blade rotation when traveling in reverse and not giving children rides (both when and when not mowing) may be critical in preventing mower-related injuries.

## Background

Annually, about 10,000 children are injured by lawn mowers with 5% resulting in amputations (Bachier and Feliz 2016; Vollman and Smith 2006). Lawn mower-related incidents are the 3rd leading cause of pediatric amputations in the U.S. (Borne et al. 2017) and the most common cause of major limb loss in children under 10 years of age (Owen 2017). One study found that over half the children treated by pediatric orthopedics for lawn mower injuries ended up with an amputation (Loder et al. 1997). The resulting impact of these injuries on the child and family can be devastating both psychologically and financially (Rusch et al. 2000; Loder et al. 2004 Dec; Weir et al. 2010). Despite increased mower safety specifications and requirements, lawn mower injury incidence rates have remained essentially unchanged for the past 40 years (Bachier and Feliz 2016; Vollman and Smith 2006; Klein et al. 2018).

Pediatric lawn mower injuries have a bimodal age distribution with peaks around 3 years and 15 years of age (Bachier and Feliz 2016; Vollman and Smith 2006). Although about a quarter of all the lawnmower injuries occur to children under the age of 5 years (Bachier and Feliz 2016; Vollman and Smith 2006), they are often more serious than those seen in older children. A study at a level 1 pediatric trauma center observed that 41% of their pediatric patients presenting for lawn mower-related injuries were under the age of 5 and that there was an inverse relationship to age and length of stay (Lee et al. 2017). Other studies have found younger children injured by lawn mowers required more complex treatment (Loder et al. 1997; Garay et al. 2017). For example, children 0–6 years had the highest mean injury severity score and seven times greater odds of intensive care unit admission as compared to older children (Garay et al. 2017). In addition, children < 5 years were six times more likely to have an amputation after a lawn mower injury than children 6 years and older (Borne et al. 2017).

Some of these serious injuries occur when lawn mower operators allow younger children to ride with them while mowing. Under many operation circumstances, it may only take a moment for the child to slip away and be run over by the mower. However, studies have shown younger children injured by riding lawnmowers have even higher proportions that are injured as a bystander (Loder et al. 1997; Shah et al. 2020), most commonly while the operator is backing up and operating the mower in reverse.

Though only 0.8% of all lawn mower-related injuries are from backups/reversing, 70% occur to children under the age of 5 (Ren et al. 2017). Most commonly, during these incidents, a young child approaches a riding lawn mower in operation and the operator turns or reverses the mower knocking over the child and running them over. Usually, the presence of the child in the area is unbeknownst to the operator whose attention is on the task at hand and is often unable to hear due to the noise of the mower and/or the use of hearing protection.

We hypothesize that children who have had a riding lawn mower injury as a bystander are likely to have been given rides on a mower prior to their injury, thus transforming these once scary machines into playthings and desensitizing children to their inherent dangers. With a desire to get a ride, children may be more likely to approach a lawn mower in use and significantly increase their chance of a traumatic lawn mower injury.

The American Academy of Pediatrics’ Committee on Injury and Poison Prevention has stated, "Additional research regarding the circumstances and contributing factors of lawn mower-related injuries is needed, especially…situations in which a person has been run over or backed over” (Bull et al. 2001). Previous studies have been limited in their ability to identify contributing risk factors and behaviors that lead to serious riding lawn mower-related injuries in children. Our study objective was to investigate the circumstances surrounding these events and specifically whether injured bystanders had been given previous lawn mower rides.

## Methods

Research team members worked with members of the University of Iowa Stead Family Children’s Hospital Injury Prevention and Community Outreach Program and with leadership from the 501c non-profit organization, Tate’s Army, to develop a survey investigating the circumstances surrounding serious lawn mower injuries in children. Tate’s Army was started by a family whose child nearly lost a leg due to a lawn mower backover. The organization’s mission is to educate, support and advocate for lawn mower and machinery safety awareness and prevention, and to provide direct financial assistance to affected families (https://tatesarmy.org/).

A collaborative and iterative process was used by the group to develop the survey. The research team was cognizant of the possible emotions that completing our survey might arouse in subjects. We were careful with our wording and frequently utilized empathic statements. The survey was administered to several families who had experienced a serious riding lawn mower injury to help validate the tool and the families provided valuable input that helped shape the survey’s final format and design. The University of Iowa Institutional Review Board approved this study.

Facebook group members of the Lawn Mower Accident (LMA) Survivors and Family Closed Support Group who have children that suffered lawnmower-related injuries (660 members) and followers of the LMA Support and Prevention Facebook page (7000 followers) were invited to complete an electronic survey. Three postings spaced apart in July 2021 were placed by the administrator of these pages with a web link to the cloud-based platform Qualtrics. Adults aware of the circumstances of a lawn mower-related injury to any child 17 years of age or less were encouraged to participate.

Multiple choice and open text questions addressed the circumstances, injuries, and behaviors before and at the time of the injury incident. Demographic questions related to the injured child and the operator of the mower involved in the incident, as well as information related to the time and place of the incident and the type of mower involved were collected. Additional questions were asked about the activities and supervision of the injured child just before the incident including who was providing supervision, how many other children were they supervising and any circumstances that may have affected the supervision provided. Description of the event, injuries sustained by the child, and treatment including surgeries required were also queried. Of particular interest, the survey addressed whether the child had received rides prior to the injury incident on a lawn mower with the blades moving (while in operation) and/or on a lawn mower with the blades not moving (not in operation).

Duplicate surveys regarding the same individual completed by multiple individuals were identified based on the month, year and state of the occurrence, demographics provided of the child, and the description of the event. Only the first complete survey submitted about a specific individual was included in the study. Surveys of incidents that did not involve a riding lawn mower, including push mowers, were removed from analysis. Descriptive statistics including frequencies and means, and comparative statistics assessing categorical variables through chi-square and Fisher exact tests were performed using the statistical software suite, SAS, version 9.4 (SAS Institute, Cary, NC). Fisher’s exact test was used for any comparison in which a cell had a predicted value of < 5. All *p* values were two-tailed and a value < 0.05 was considered statistically significant. Missing data were not included in analyses.

## Results

Surveys were completed by participants regarding 140 children injured on riding lawn mowers. Over four-fifths (83%, 115/139) of the injuries described occurred since the year 2000 and two-thirds (66%, 92/139) since the year 2010 (data not shown). Most respondents (71%) were parents of the injured child but almost one-fifth were an adult survivor of a childhood injury (Table 1). The children’s ages ranged from 12 months to 16 years of age with 57% being 2–4 years old at the time of their injury. Most (64%) were male, and the vast majority (94%) were non-Hispanic white children. Over half had insurance through a parent’s employer and one-quarter were covered by Medicare/Medicaid. Over half (54%) stated the child lived in the country but not on a farm/ranch at the time of the injury, while almost 40% lived in a town or city. The majority of injured children (73%) lived in the Midwest or South census regions. Nearly 30% were an only child in their family at the time of the injury; about 40% had 2 or more siblings.

**Table 1:** Frequencies of study demographic variables regarding children injured during riding lawn mower incidents as reported on surveys completed by followers of the Lawn Mower Accident (LMA) Support and Prevention Facebook page and members of the LMA Survivors and Family Closed Support Group on Facebook (N = 140)

Variable	n (Col%)^a, b^
*Person completing survey*	
Parent of injured child	100 (71)
Adult survivor of childhood injury	27 (19)
Grandparent of injured child	7 (5)
Other relative of injured child	4 (3)
Family friend/neighbor of injured child	1 (1)
First responder/good samaritan	1 (1)
*Age*	
< 1 yr	0 (0)
1 yr	8 (6)
2 yrs	23 (16)
3 yrs	32 (23)
4 yrs	25 (18)
5 yrs	19 (14)
6–7 yrs	19 (14)
8–9 yrs	6 (4)
10–16 yrs	8 (6)
*Sex/gender*	
Male	89 (64)
Female	50 (36)
Non-binary/third gender	1 (1)
*Race/ethnicity*	
Non-Hispanic White	131 (94)
Hispanic/Latinx	4 (3)
Black or African American	1 (1)
Asian	2 (1)
Other	2 (1)
*Insurance*	
Medicare/Medicaid	35 (25)
Through an employer	74 (53)
Private	6 (4)
No insurance	6 (4)
Don’t know/missing	19 (14)
*Where they lived*	
Farm/ranch	8 (6)
In the country/not on a farm or ranch	76 (54)
Town/city	56 (40)
*Census region*	
Northeast	27 (19)
Midwest	47 (34)
South	55 (39)
West	5 (4)
Country other than U.S	6 (4)
*Number of other children in family*	
0	41 (29)
1	43 (31)
2	29 (21)
≥ 3	27 (19)

*Col%*—column percent; *mos*—months; *n*—number in variable subgroup; *yrs*—years

^a^Column total may not equal group N due to missing data

^b^Total column percentage may not equal 100% due to rounding

**Fig. 1:**
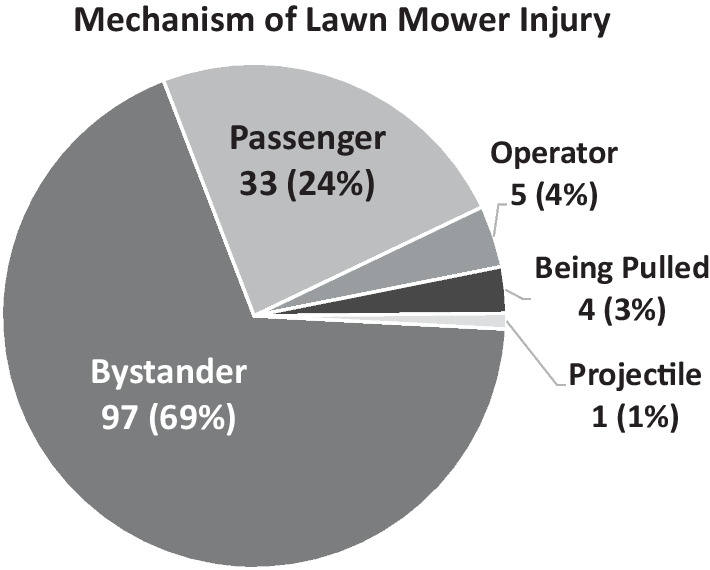
Mechanism of lawn mower injury. The proportion of children injured by various mechanisms related to riding lawn mowers as reported on surveys completed by followers of the Lawn Mower Accident (LMA) Support and Prevention Facebook page and members of the LMA Survivors and Family Closed Support Group on Facebook. Mechanisms include being injured as a bystander not involved in the mowing, as a passenger on the mower before being injured, as a mower operator, while being pulled in a trailer or wagon by the mower, and being struck by a projectile

The majority of children (69%) were injured as a bystander that was not involved in the mowing (Fig. 1). About a quarter (24%) of the children were riding on the mower as a passenger before being injured. Smaller proportions were injured while operating the mower, struck by a projectile or while being pulled in a trailer or wagon by the mower.

Most injuries (80%) occurred in the spring and summer months, and over half took place between 2 and 6 pm (Table 2). The vast majority (79%) transpired on the lawn of the family’s home with another 15% occurring on a relative’s property. The mower operator was most often male (77%) with a father/stepfather (45%) or grandfather (13%) being most frequent. In 59% of cases, the mower was traveling in reverse at the time of the incident.

**Table 2:** Frequencies of study injury event variables regarding children injured during riding lawn mower incidents as reported on surveys completed by followers of the Lawn Mower Accident (LMA) Support and Prevention Facebook page and members of the LMA Survivors and Family Closed Support Group on Facebook (*N* = 140)

Variable	*n* (Col%)^a, b^
*Season event occurred*	
Spring (Mar–May)	51 (36)
Summer (Jun–Aug)	62 (44)
Fall (Sept–Nov)	26 (19)
Winter (Dec–Feb)	1 (1)
*Time event occurred*^*c*^	
6:00–9:59 am	4 (3)
10:00 am–1:59 pm	42 (31)
2:00–5:59 pm	70 (52)
6:00–9:59 pm	19 (14)
*Place event took place*	
Home lawn	111 (79)
Relative’s lawn	21 (15)
Neighbor’s lawn	6 (4)
Other	2 (1)
*Operator sex*	
Male	103 (77)
Female	30 (22)
Non-binary/third gender	1 (1)
*Mower operator relation to injured child*	
Father/stepfather	63 (45)
Mother/stepmother	18 (13)
Grandfather	18 (13)
Grandmother	5 (4)
Sibling	11 (8)
Other relative	11 (8)
Child that was injured	6 (4)
Other	8 (6)
*Direction of travel*^*c*^	
Forward	35 (29)
Turning right or left	10 (8)
Reverse/backwards	72 (59)
Other (rollover, collision, stationary)	5 (4)
*Supervisor of child just before injury*^*c*^	
Father/stepfather	53 (38)
Mother/stepmother	43 (31)
Grandfather/grandmother	20 (14)
Multiple	5 (4)
No one designated to supervise	10 (7)
Other	9 (6)

*Col%*—column percent; *n*—number in variable subgroup

^a^Column total may not equal group N due to missing data

^b^Total column percentage may not equal 100% due to rounding

^c^Those that answered “I Don’t Know/Not Sure” not included

A variety of individuals were reported as being the one responsible for supervising the child at the time of the injury. This was most frequently the father/stepfather (38%) or the mother/stepmother (31%) of the child. Others responsible for supervision included grandparents (14%), multiple individuals (4%) and no one (7%). Identified individuals were responsible for supervising an average of 1.1 children in addition to the child that was injured at the time of the incident with a range of 0–15 additional children.

Of the 91% of respondents who were aware of clinical outcomes, 99% (126/127) stated the injured child was hospitalized (Table 3). Hospital length of stay ranged from 1 to 100 days with a mean stay of 26 days. Nearly two-thirds (65%) were admitted to the intensive care unit with time spent there ranging from 1 to 35 days with an average length of stay of 8.6 days. Almost all children (98%) required surgery. The total number of surgeries children had undergone since their injury (108 subjects reporting) ranged from 1 to 37 with an average of 7.3 surgeries. Over three-quarters of children suffered an amputation with 92% of survey respondents reporting the child had incurred at least one amputation and/or permanent disability.

**Table 3:** Frequencies of study injury variables regarding clinical outcomes of children injured during riding lawn mower incidents as reported on surveys completed by followers of the Lawn Mower Accident (LMA) Support and Prevention Facebook page and members of the LMA Survivors and Family Closed Support Group on Facebook (*N* = 140)

Variable	*n* (Col%)^a^
*Hospitalized*	
Yes	126 (99)
No	1 (1)
*Admitted to intensive care unit*^*b*^	
Yes	76 (65)
No	41 (35)
*Required surgery*	
Yes	124 (98)
No	2 (2)
*Amputation*^*b*^	
Yes	101 (76)
No	32 (24)
*Amputation and/or permanent disability*^*b*^	
Yes	115 (92)
No	10 (8)

*Col%*—Column percent; *n*—number in variable subgroup

^a^Column total may not equal group N due to missing data

^b^Those that answered “I Don’t Know/Not Sure” not included

Table 4 shows the sub-analysis of those injured as bystanders as compared to passengers. A significantly higher proportion of injured bystanders (71%) were < 5 years of age as compared to injured passengers (45%), *p* = 0.01. A higher proportion of bystanders (77%) were injured when the lawn mower’s direction of travel was in reverse as compared to passengers (17%), *p* < 0.01. Most passengers were injured while the mower was moving forward (66%). Amputations and/or permanent disabilities were greater among bystanders as compared to passengers (97% vs. 79%, respectively; *p* = 0.01). Only 3% (6/97) bystanders had an injury to their upper extremity as compared to 21% (7/33) of passengers, *p* < 0.01 (data not shown). A higher proportion of bystanders (67%, 65/97) had injuries to the lower extremities as compared to passengers (45%, 15/33), *p* = 0.03.

**Table 4:** Comparison of study variables regarding children injured during riding lawn mower incidents while riding as a passenger and as a bystander not involved in the mowing as reported on surveys completed by followers of the Lawn Mower Accident (LMA) Support and Prevention Facebook page and members of the LMA Survivors and Family Closed Support Group on Facebook

Variable	Passengers n (Col%)^a, b^	Bystanders n (Col%)^a, b^	*p* Value
*Total*	33	97	
*Age*			0.01
< 3 yrs	8 (24)	21 (22)	
3–4 yrs	7 (21)	48 (49)	
≥ 5 yrs	18 (55)	28 (29)	
*Race/ethnicity*			0.10
Non-Hispanic White	29 (91)	92 (98)	
Other	3 (9)	2 (2)	
*Insurance*			0.36
Medicare/Medicaid	5 (19)	27 (34)	
Through an employer	19 (73)	49 (61)	
Private	2 (8)	4 (5)	
*Where they lived*			0.18
Farm/ranch	2 (6)	5 (5)	
In the country/not on a farm or ranch	13 (39)	56 (58)	
Town/city	18 (55)	36 (37)	
*Census region*			0.27
Northeast	6 (18)	16 (16)	
Midwest	8 (24)	38 (39)	
South	17 (52)	35 (36)	
West	2 (6)	3 (3)	
*Number of children in family*			0.37
1	11 (33)	26 (27)	
2	7 (21)	32 (33)	
3	10 (43)	19 (20)	
≥ 4	5 (15)	20 (21)	
*Season of the year*			0.76
Spring	12 (36)	36 (37)	
Summer	13 (39)	43 (44)	
Fall	8 (24)	18 (19)	
*Time event occurred*^c^			0.22
6 am-2:59 pm	18 (56)	41 (44)	
3 pm-10 pm	14 (44)	53 (56)	
*Place event took place*			0.94
Home	26 (79)	77 (79)	
Other	7 (21)	20 (21)	
*Operator relation to injured child*			0.62
Father/stepfather	14 (42)	46 (47)	
Other	19 (58)	51 (53)	
*Operator sex*			0.63
Male	25 (76)	73 (75)	
Female	7 (21)	24 (25)	
Other	1 (3)	0 (0)	
*Direction of travel*^c^			< 0.01
Forward	19 (66)	14 (17)	
Turning to left or to right	5 (17)	5 (6)	
Reverse	5 (17)	62 (77)	
*Admitted to ICU*^c^			0.08
Yes	15 (52)	57 (70)	
No	14 (48)	25 (30)	
*Required surgery*^c^			0.07
Yes	29 (94)	88 (100)	
No	2 (6)	0 (0)	
*Amputation*^c^			0.41
Yes	22 (71)	72 (78)	
No	9 (29)	20 (22)	
*Amputation and/or permanent disability*^c^			0.01
Yes	23 (79)	85 (97)	
No	6 (21)	3 (3)	

*Col%*—column percent; *n*—number in variable subgroup; *yrs*—years

^a^Column total may not equal group N due to missing data

^b^Total column percentage may not equal 100% due to rounding

^c^Those that answered “I Don’t Know/Not Sure” not included

A substantial number of children injured as bystanders had received prior rides on a riding lawn mower. Of participants who provided a “Yes” or “No” response for the following variables, 57% (50/87) stated the injured bystander had received prior rides on a lawn mower while the blades were in operation and 60% (44/73) when the blades were not in operation. Combining these categories, 73% (61/84) of children had received a ride on a riding lawn mower prior to their injury as a bystander. The number of rides children had received prior to their injury as a bystander ranged from 1 to 100 with many respondents simply stating “multiple” or “a lot”.

## Discussion

In this study, we investigated pediatric riding lawn mower-related injuries by surveying followers of a lawn mower injury support and prevention Facebook page, and members of a Facebook support group who are survivors or family members of children injured by lawn mowers. The overall age of injured children was quite young with over three-quarters being 5 years of age or less. The vast majority suffered an amputation and/or other permanent disability as a result of their injury. Most injuries occurred as children were bystanders not involved with the mowing. There were significant differences noted between injured bystanders and passengers with bystanders having higher proportions being younger, injured while the mower was in reverse, and having incurred an amputation and/or permanent disability. Seventy-three percent of respondents reported that injured bystanders had received rides on a lawn mower prior to the incident.

Most of the injuries reported were from the Midwest and South. This is consistent with a recent study of lawn mower injuries in the Pediatric Health Information System database where incidence rates per 100,0000 patient encounters at 49 children’s hospitals were 2.70 and 2.16 in the Midwest and South as compared to 1.34 and 0.56 in the Northeast and West, respectively (Shah et al. 2020). Virtually all children in the study were hospitalized for their injury. The proportion of lawn mower-related injuries requiring hospitalization is twofold greater than that for other consumer product-related injuries overall (Vollman and Smith 2006).

About a quarter of respondents provided information on their survey about a child injured while riding as a passenger on a riding lawn mower that was in operation. There are no seats for passengers on a riding lawn mower. Respondents anecdotally reported child passengers were either standing on the back of the mower deck or hitch while holding onto the seat, or more typically sitting on the lap of the operator or on the mower deck between the operator’s feet when these injury events occurred. In some of the cases, the child purposefully jumped off (usually not knowingly to the operator) and slid under the deck and into the moving blades. In most of the cases, the child slipped or fell off, sometimes while the mower was turning, but usually because of uneven or rough terrain such as hitting a hole or bump. In one case, the child reached down to try and retrieve a shoe that had fallen off. In several instances, multiple children were riding as passengers. Almost all safety guidelines state children should never be allowed to ride as passengers on riding lawn mowers (Bull et al. 2001; Academy and of Orthopaedic Surgeons. Power Lawnmower Safety. 1142; University of Iowa Stead Family Children’s Hospital. Lawn mower safety tips: patient education 2016).

The most common mechanism of injury reported in the survey was that of a bystander slipping under or being knocked over and then being cut by the mower’s rotary blades. Anecdotally, respondents in some cases reported the child was known to be playing or doing activities near the mowing location. In a few of these cases, the child slipped and fell, for example, after going down a slide or retrieving a football, and ended up with an extremity under the operating mower. However, most bystander injuries involved the child running up to or trying to jump onto a riding mower unbeknownst to the operator. Often, at the time of injury, the child was approaching the lawn mower from behind and the operator was just backing up the lawn mower, which probably facilitated the child catching up to the mower. The child then slipped or was knocked over by the reversing mower and run over with the blades in operation. In our study, the lawn mower was traveling backwards at the time of the injury in over three-quarters of bystander cases.

A 2003 update in the voluntary safety standard, ANSI/OPEI B71.1, prohibits all riding lawn mowers manufactured after September 1, 2004, from mowing continually after the operator shifts into reverse (MTD 2017). This safety measure was primarily implemented to decrease the number of children seriously injured by mowers as bystanders (U.S. CPSC 2015). However, most lawn mowers built since that time can still mow in reverse using an override system (U.S. CPSC 2015). The method to accomplish this varies among mowers but includes having to push a button before the transmission is shifted into reverse, pressing a button the entire time you want to mow in reverse, or turning the key to the reverse position and then repositioning the key again when you want to move forward (Dooley 2021; Gerhardt 2023). Moreover, zero turn radius and front mount mowers are excluded from the requirement (U.S. CPSC 2015), and there are likely hundreds of thousands of older riding mowers that do not have any “no mow in reverse” safety feature (Kitzes 2001).

For backover cases in the study, respondents specifically stated that the mower did not have a “no mow in reverse” feature in nine cases and for eight of the cases the feature had been deactivated. This may have been the situation for other cases in the study as well but not voluntarily provided. Ideally, to protect children from amputations and other serious injuries, all riding lawn mowers would not be able to mow in reverse. Manufacturers and the U.S. Consumer Product Safety Commission could at least help reduce the risk by requiring placement of the override button behind operators so they would have to turn back to engage it, allowing them to see if there’s a child nearby (Academy and of Orthopaedic Surgeons 2022).

Most safety guidelines recommend that young children must not be allowed to play in or be adjacent to areas where lawn mowers are in operation and should be kept indoors during mowing (Bull et al. 2001 Jun; University of Iowa Stead Family Children’s Hospital 2016; Prevent Child Injury 2023). This requires, for many children, a significant level of supervision. In the study, individuals supervising injured bystanders were responsible, on average, for at least one other child and supervising up to fifteen other children. Supervisor distraction often played an important factor in many cases. For example, responsibilities to another child sometimes took precedence or they were distracted by other activities such as carrying groceries inside or conversation with another adult. Often, respondents stated the lack of attention to the child was so brief and that it happened so fast that they could not respond in time to stop it from happening. In a number of cases, the mower operator was stated as being the one supervising the child. Obviously, this is not adequate child supervision. We did not ask, and it is unclear if the person reported as supervising the child was always aware that they were the one responsible for such duties.

We believe that allowing children to be passengers on a riding lawn mower is not only a danger while the mower blades are in operation but at other times as well. Our hypothesis is that giving lawn mower rides acclimates young children to the loud noises and vibrations of mowers, desensitizes them to their inherent dangers, and converts mowers from working machinery to a riding plaything in children’s minds. Many of the respondents stated in the survey that the child injured as a bystander was approaching the lawnmower to get a ride, and it is likely that this was the intention of many other child bystanders as well.

Over half (57%) of injured bystanders in the study had been given a ride on a riding lawn mower with the blades in operation. In some of the cases, the child had been riding on the operating mower earlier on the day of injury. However, there are many parents, grandparents and other individuals that would never have a child as a passenger on a riding lawn mower with the blades in motion but will give a ride to a young child when the blades are not rotating. In fact, that was true for 15% of the bystander victims in the study and increased the total proportion of injured bystanders having received a prior ride on a riding lawn mower to 73%. Family members often find this to be a fun and bonding activity with the young children they love. Unfortunately, it may also lead to extremity amputations requiring numerous surgeries and permanent disability.

### Limitations

The study was limited to followers of the LMA Support and Prevention Facebook page and Facebook group members of the LMA Survivors and Family Closed Support Group so the information collected was meant to be partial towards children with more serious injuries and is not representative of all lawn mower injuries in children. Affected individuals and families who do not participate in social media or were not followers of this specific Facebook page would not have been included. As survey information was anonymously self-reported, study data cannot be confirmed or denied. Since many were describing an incident that happened some years in the past, our participants were likely at risk for recall bias.

## Conclusions

Children injured by riding lawn mowers often suffer very serious injuries causing permanent disability including amputations. Young children are frequently injured when as a passenger they fall or jump off with an extremity ending up under the mower. However, they are even more commonly injured as a bystander when approaching the mower–often at the time the operator is backing up. We found child bystanders seriously injured by riding lawn mowers were frequently given prior rides. This likely acclimates the child to the machine and converts it into a plaything in the child’s mind leading to the child seeking rides when the mower is in operation. Engineering changes to prevent mower blade rotation when traveling in reverse and not giving children rides on lawn mowers (both when and when not mowing) may be critical in preventing these serious injuries.

## Data Availability

Data and materials are available to other parties for research purposes after a data sharing agreement plan is agreed to and signed. Those interested should contact the corresponding author.

## Abbreviations

ANSI: American National Standards Institute
LMA: Lawn mower accident
NC: North Carolina
OPEI: Outdoor Power Equipment Institute
SAS: Previously Statistical Analysis System
U.S.: United States
